# Tinted, Detached, and Lazy CNF-XOR Solving and Its Applications to Counting and Sampling

**DOI:** 10.1007/978-3-030-53288-8_22

**Published:** 2020-06-13

**Authors:** Mate Soos, Stephan Gocht, Kuldeep S. Meel

**Affiliations:** 8 Microsoft Research Lab, Redmond, WA USA; 9 University of Southern California, Los Angeles, CA USA; 10 School of Computing, National University of Singapore, Singapore, Singapore; 11 Lund University, Lund, Sweden

## Abstract

Given a Boolean formula, the problem of counting seeks to estimate the number of solutions of *F* while the problem of uniform sampling seeks to sample solutions uniformly at random. Counting and uniform sampling are fundamental problems in computer science with a wide range of applications ranging from constrained random simulation, probabilistic inference to network reliability and beyond. The past few years have witnessed the rise of hashing-based approaches that use XOR-based hashing and employ SAT solvers to solve the resulting CNF formulas conjuncted with XOR constraints. Since over 99% of the runtime of hashing-based techniques is spent inside the SAT queries, improving CNF-XOR solvers has emerged as a key challenge.

In this paper, we identify the key performance bottlenecks in the recently proposed $\mathsf {BIRD}$ architecture, and we focus on overcoming these bottlenecks by accelerating the XOR handling within the SAT solver and on improving the solver integration through a smarter use of (partial) solutions. We integrate the resulting system, called $\mathsf {BIRD2}$, with the state of the art approximate model counter, $\mathsf {ApproxMC3}$, and the state of the art almost-uniform model sampler $\mathsf {UniGen2}$. Through an extensive evaluation over a large benchmark set of over 1896 instances, we observe that $\mathsf {BIRD2}$ leads to consistent speed up for both counting and sampling, and in particular, we solve 77 and 51 more instances for counting and sampling respectively.

## Introduction

A CNF-XOR formula $\varphi$ is represented as conjunction of two Boolean formulas $\varphi _{\text {CNF}} \wedge \varphi _{\text {XOR}}$ wherein $\varphi _{CNF}$ is represented in Conjunctive Normal Form (CNF) and $\varphi _{XOR}$ is represented as conjunction of XOR constraints. While owing to the NP-completeness of CNF, every CNF-XOR formula can be represented as a CNF formula with only a linear increase in the size of the resulting formula, such a transformation may not be ideal in several scenarios. In particular, it is well known that modern Conflict Driven Clause Learning (CDCL) SAT solvers perform poorly on XOR formulas represented in CNF form despite the existence of efficient polynomial time decision procedures for XOR constraints. Furthermore, constraints arising from domains such as cryptanalysis and circuits can be naturally described as CNF-XOR formulas and these domains served as the early inspiration for design of SAT solvers with native support for XORs through the usage of Gaussian Elimination. These efforts lead to the development of $\mathsf {CryptoMiniSat}$, a SAT solver that sought to perform Conflict Driven Clause Learning and Gaussian Elimination in tandem. The architecture of the early verisons of $\mathsf {CryptoMiniSat}$ sought to employ disjoint storage of CNF and XOR clauses – reminiscent to the architecture of SMT solvers.

While $\mathsf {CryptoMiniSat}$ was originally designed for cryptanalysis, its ability to handle XORs natively has led it to be a fundamental building block of the hashing-based techniques for approximate model counting and sampling. Model counting, also known as #SAT, and uniform sampling of solutions for Boolean formulas are two fundamental problems in computer science with a wide variety of applications [1, 11, 18]. The core idea of hashing-based techniques for approximate counting and almost-uniform sampling is to employ XOR-based 3-wise independent hash functions^1^ to partition the solution space of *F* into *roughly equal small* cells of solutions. The usage of XOR-based hash functions allows us to represent a cell as conjunction of a Boolean formula in conjunctive normal form (CNF) and XOR constraints, and a SAT solver is invoked to enumerate solutions inside a randomly chosen cell. The corresponding counting and sampling algorithms typically employ the underlying solver in an incremental fashion and invoke the solver thousands of times, thereby necessitating the need for runtime efficiency. In this context, Soos and Meel [19] observed that the original architecture of $\mathsf {CryptoMiniSat}$ did not allow a straightforward integration of pre- and in-processing which of late has emerged to be key techniques in SAT solving. Accordingly, Soos and Meel [19] proposed a new architecture, called $\mathsf {BIRD}$, that relied on the key idea of keeping the XOR constraints in both CNF form and XOR form. Soos and Meel integrated $\mathsf {BIRD}$ into $\mathsf {CryptoMiniSat}$, and showed that state of the art approximate model counter, $\mathsf {ApproxMC}$, when integrated with the new version of $\mathsf {CryptoMiniSat}$ achieves significant runtime improvements. The resulting version of $\mathsf {ApproxMC}$ was called $\mathsf {ApproxMC3}$.

Motivated by the success of $\mathsf {BIRD}$ in achieving significant runtime performance improvements, we sought to investigate the key bottlenecks in the runtime performance of $\mathsf {CryptoMiniSat}$ when handling CNF+XOR formulas. Given the prominent usage of CNF-XOR formulas by the hashing based techniques, we study the runtime behavior of $\mathsf {CryptoMiniSat}$ for the the queries issued by the hashing-based approximate counters and samplers, $\mathsf {ApproxMC3}$ and $\mathsf {UniGen2}$ respectively. Our investigation leads us to make five core technical contributions. The first four contributions contribute towards architectural advances in handling of CNF-XOR formulas while the fifth contribution focuses on algorithmic improvements in the hashing-based techniques for counting and sampling:

1. **Matrix row handling improvements** for efficient propagation and conflict checking of XOR constraints
2. **XOR constraint detaching** from the standard unit propagation system for higher unit propagation speed
3. **Lazy reason clause generation** to reduce reason generation overhead for unused reasons generated from XOR constraints
4. **Allowing partial solution extraction** by the SAT solver
5. **Intelligent reuse of solutions** by hashing-based techniques to reduce the number of SAT calls

We integrate these improvements into the $\mathsf {BIRD}$ framework, the resulting framework is called $\mathsf {BIRD2}$. The $\mathsf {BIRD2}$ framework is applied to state of the art approximate model counter, $\mathsf {ApproxMC3}$, and to the almost-uniform sampler $\mathsf {UniGen2}$ [6, 9]. The resulting counter and sampler are called $\mathsf {ApproxMC4}$ and $\mathsf {UniGen3}$ respectively. We conducted an extensive empirical evaluation with over 1800 benchmarks arising from diverse domains with computational effort totalling 50,000 CPU hours. With a timeout of 5000 s, $\mathsf {ApproxMC3}$ and $\mathsf {UniGen2}$+$\mathsf {BIRD}$ were able to solve only 1148 and 1012 benchmarks, while $\mathsf {ApproxMC4}$ and $\mathsf {UniGen3}$ solved 1225 and 1063 benchmarks respectively. Furthermore, we observe a consistent speedup for most of the benchmarks that could be solved by $\mathsf {ApproxMC3}$ and $\mathsf {UniGen2}$+$\mathsf {BIRD}$. In particular, the PAR-2^2^ score improved from 4146 with $\mathsf {ApproxMC3}$ to 3701 with $\mathsf {ApproxMC4}$. Similarly, the corresponding PAR-2 scores for $\mathsf {UniGen3}$ and $\mathsf {UniGen2}$+$\mathsf {BIRD}$ improved to 4574 from 4878.

## Notations and Preliminaries

Let *F* be a Boolean formula in conjunctive normal form (CNF) and $\mathsf {Vars }(F)$ the set of variables in *F*. Unless otherwise stated, we use *n* to denote the number of variables in *F* i.e., $n=|\mathsf {Vars }(F)|$. An assignment of truth values to the variables in $\mathsf {Vars }(F)$ is called a *satisfying assignment* or *witness* of *F* if it makes *F* evaluate to true. We denote the set of all witnesses of *F* by $sol({F})$. If we are only interested in a subset of variables $S \subseteq \mathsf {Vars }(F)$ we will use $sol({F})_{\downarrow S}$ to indicate the projection of $sol({F})$ on *S*.

The problem of *propositional model counting* is to compute $|sol({F})|$ for a given CNF formula *F*. A *probably approximately correct* (or $\mathsf {PAC}$) counter is a probabilistic algorithm ${\mathsf {ApproxCount}}(\cdot , \cdot ,\cdot )$ that takes as inputs a formula *F*, a tolerance $\varepsilon >0$, and a confidence $1-\delta \in (0, 1]$, and returns a count *c* with $(\varepsilon ,\delta )$-guarantees, i.e., $\mathsf {Pr}\Big [|sol({F})|/(1+\varepsilon ) \le c \le (1+\varepsilon )|sol({F})|\Big ] \ge 1-\delta$. Projected model counting is defined analogously using $sol({F})_{\downarrow S}$ instead of $sol({F})$, for a given sampling set $S \subseteq \mathsf {Vars }(F)$.

A *uniform sampler* outputs a solution $y \in sol({F})$ such that $\mathsf {Pr}[y \text { is output}] = \frac{1}{|sol({F})|}$. An *almost-uniform sampler* relaxes the guarantee of uniformity and in particular, ensures that $\frac{1}{(1+\varepsilon )|sol({F})|} \le \mathsf {Pr}[y \text { is output}] \le \frac{1+\varepsilon }{|sol({F})|}$.

**Universal Hash Functions.** Let $n,m\in \mathbb {N}$ and $\mathcal {H}(n,m) \triangleq \{ h:\{0,1\}^{n} \rightarrow \{0,1\}^m \}$ be a family of hash functions mapping $\{0,1\}^n$ to $\{0,1\}^m$. We use $h \xleftarrow {R} \mathcal {H}(n,m)$ to denote the probability space obtained by choosing a function *h* uniformly at random from $\mathcal {H}(n,m)$. To measure the quality of a hash function we are interested in the set of elements of *S* mapped to $\mathbf {\alpha }$ by *h*, denoted $\mathsf {Cell}_{\langle S, h, \mathbf {\alpha } \rangle }$ and its cardinality, i.e., $|\mathsf {Cell}_{\langle S, h, \mathbf {\alpha } \rangle }|$. To avoid cumbersome terminology, we abuse notation slightly and we use $\mathsf {Cell}_{\langle F, m \rangle }$ (resp. $\mathsf {Cnt}_{\langle F, m \rangle }$) as shorthand for $\mathsf {Cell}_{\langle sol({F}), h, \mathbf {\alpha } \rangle }$ (resp. $|\mathsf {Cell}_{\langle sol({F}), h, \mathbf {\alpha } \rangle }|$).

### Definition 1

A family of hash functions $\mathcal {H}(n,m)$ is k-wise independent^3^ if $\forall \mathbf {\alpha }_1, \mathbf {\alpha }_2, \ldots \mathbf {\alpha }_k \in \{0,1\}^m$ and for distinct $\mathbf {y}_1, \mathbf {y}_2, \ldots \mathbf {y}_k \in \{0,1\}^n$, $h \xleftarrow {R} \mathcal {H}(n,m)$,

1 $$\begin{aligned} \mathsf {Pr}\left[ (h(\mathbf {y}_1) = \mathbf {\alpha }_1) \wedge ( h(\mathbf {y}_2) = \mathbf {\alpha }_2) \ldots \wedge (h(\mathbf {y}_k) = \mathbf {\alpha }_k) \right] = \left( \frac{1}{2^m}\right) ^k \end{aligned}$$

Note that every *k*-wise independent hash family is also $k-1$ wise independent.

**Prefix Slicing.** While universal hash families have nice concentration bounds, they are not adaptive, in the sense that one cannot build on previous queries. In several applications of hashing, the dependence between different queries can be exploited to extract improvements in theoretical complexity and runtime performance. Thus, we are typically interested in prefix slices of hash functions [10] as follows.

### Definition 2

For every $m \in \{1, \ldots n\}$, the $m^{th}$ prefix-slice of *h*, denoted $h^{(m)}$, is a map from $\{0,1\}^{n}$ to $\{0,1\}^m$, such that $h^{(m)}(\mathbf {y})[i] = h(\mathbf {y})[i]$, for all $y \in \{0,1\}^{n}$ and for all $i \in \{1, \ldots m\}$. Similarly, the $m^{th}$ prefix-slice of $\alpha$, denoted $\alpha ^{(m)}$, is an element of $\{0,1\}^m$ such that $\alpha ^{(m)}[i] = \alpha [i]$ for all $i \in \{1, \ldots m\}$.

**Explicit Hash Functions.** The most common explicit hash family used in state of the art sampling and counting techniques is based on random XOR constraints. Viewing $\mathsf {Vars }(F)$ as a vector $\textit{\textbf{ x }}$ of dimension $n \times 1$, we can represent the hash family as follows: Let $\mathcal {H}_{xor}(n,m) \triangleq \{ h:\{0,1\}^{n} \rightarrow \{0,1\}^m \}$ be the family of functions of the form $h(x) = \textit{\textbf{ M }}\textit{\textbf{ x }}+\textit{\textbf{ b }}$ with $\textit{\textbf{ M }} \in \mathbb {F}_{2}^{m \times n}$ and $\textit{\textbf{ b }} \in \mathbb {F}_{2}^{m \times 1}$ where the entries of $\textit{\textbf{ M }}$ and *b* are independently generated according to the Bernoulli distribution with probability 1/2. Observe that $h^{(m)}(x)$ can be written as $h^{(m)}(\textit{\textbf{ x }})= \textit{\textbf{ M }}^{(m)}\textit{\textbf{ x }}+\textit{\textbf{ b }}^{(m)}$, where $\textit{\textbf{ M }}^{(m)}$ denotes the submatrix formed by the first *m* rows and *n* columns of $\textit{\textbf{ M }}$ and $\textit{\textbf{ b }}^{(m)}$ is the first *m* entries of the vector $\textit{\textbf{ b }}$. It is well known that $\mathcal {H}_{xor}$ is 3-wise independent [9].

## Background

The general idea of hashing-based model counting and sampling is to use a hash function from a suitable family, e.g. $\mathcal {H}_{xor}$, to divide the solution space into cells that are sufficiently small such that all solutions within a cell can be enumerated efficiently. Given such a cell, its size can then be used to estimate the total count of solutions or we can return a random element of this small cell to produce a sample. Hence, hashing-based sampling and counting are closely related.

### Hashing-Based Model Counting

The seminal work of Valiant [24] established that #SAT is #P-complete. Toda [22] showed that the entire polynomial hierarchy is contained inside the complexity class defined by a polynomial time Turing machine equipped with #P oracle. Building on Carter and Wegman’s [4] seminal work of universal hash functions, Stockmeyer [21] proposed a probabilistic polynomial time procedure relative to an NP oracle to obtain an $(\varepsilon ,\delta )$-approximation of *F*.

The core theoretical idea of the hashing-based approximate solution counting framework proposed in $\mathsf {ApproxMC}$ [8], building on Stockmeyer [21], is to employ 2-universal hash functions to partition the solution space, denoted by $sol({F})$ for a formula *F*, into *roughly equal small* cells, wherein a cell is called *small* if it has solutions less than or equal to a pre-computed threshold, $\mathsf {thresh}$. An $\mathsf {NP}$ oracle is employed to check if a cell is small by enumerating solutions one-by-one until either there are no more solutions or we have already enumerated $\mathsf {thresh}+1$ solutions. In practice, a SAT solver is used to realize the $\mathsf {NP}$ oracle. To ensure polynomially many calls to the oracle, $\mathsf {thresh}$ is set to be polynomial in the input parameter $\varepsilon$. To determine the right number of cells, i.e., the value of *m* for $\mathcal {H}(n,m)$, a search procedure is invoked. Finally, the subroutine, called $\mathsf {ApproxMCCore}$, computes the estimate as the number of solutions in the randomly chosen cell scaled by the number of cells (i.e, $2^m$). To achieve probabilistic amplification of the confidence, multiple invocations of the underlying subroutine, $\mathsf {ApproxMCCore}$, are performed with the final count computed as the median of estimates returned by $\mathsf {ApproxMCCore}$.

Two key algorithmic improvements proposed in $\mathsf {ApproxMC2}$ [10] are significant to practical performance: (1) the search for the right number of cells can be performed via galloping search, and (2) one can first perform linear search over a small enough interval (chosen to be of size 7) around the value of *m* found in the previous iteration of $\mathsf {ApproxMCCore}$. The practical profiling of $\mathsf {ApproxMC2}$ reveals that linear search is sufficient after the first invocation of $\mathsf {ApproxMCCore}$. Note that the linear search seeks to identify a value of *m* such that $\mathsf {Cnt}_{\langle F, m-1 \rangle } \ge \mathsf {thresh}$ and $\mathsf {Cnt}_{\langle F, m \rangle } < \mathsf {thresh}$ for an appropriately chosen $\mathsf {thresh}$. $\mathsf {ApproxMC}$ is currently in its third generation: $\mathsf {ApproxMC3}$.

### Hashing-Based Sampling

Jerrum, Valiant, and Vazirani [14] showed that the approximate counting and almost-uniform counting are polynomially inter-reducible. Building on Jerrum et al.’s result, Bellare, Goldreich, and Petrank [2] proposed a probabilistic uniform generator that makes polynomially many calls to an NP oracle where each NP query is the input formula *F* conjuncted with constraints encoding a degree *n* polynomially representing *n*-wise independent hash functions where *n* is the number of variables in *F*. The practical implementation of Bellare et al.’s technique did not scale beyond few tens of variables. Chakraborty, Meel, and Vardi [7, 9], sought to combine the inter-reducibility and the usage of independent hashing, and proposed a hashing-based framework, called $\mathsf {UniGen}$, that employs 3-wise independent hashing and makes polynomially many calls to an NP oracle.

The core theoretical idea of the hashing-based sampling framework, proposed in $\mathsf {UniGen}$, exploits the close relationship between counting and sampling. $\mathsf {UniGen}$ first invokes $\mathsf {ApproxMC}$ to compute an estimate of the number of solutions of the given formula *F*. It then uses the count to determine the number of cells that the solution space should be partitioned into using 3-wise independent hash functions. At this point, it is worth mentioning that the state of the art hashing-based sampling employ 3-wise independent hash functions. Fortunately, the family of hash functions, $\mathcal {H}_{xor}$, is also known to be 3-wise independent. There after, similar to $\mathsf {ApproxMC}$, a linear search over a small enough interval (chosen to be of size 4) is invoked to find the *right* value of *m* where a randomly chosen cell’s size is within the desired bounds. For such a cell, all its solutions are enumerated and one of the solutions is randomly chosen. Again, similar to $\mathsf {ApproxMC2}$ (and $\mathsf {ApproxMC3}$), the linear search seeks to identify a value of *m* such that $\mathsf {Cnt}_{\langle F, m-1 \rangle } \ge \mathsf {thresh}$ and $\mathsf {Cnt}_{\langle F, m \rangle } < \mathsf {thresh}$ for an appropriately chosen $\mathsf {thresh}$. $\mathsf {UniGen}$ is currently in its second generation: $\mathsf {UniGen2}$ [6].

### The Underlying SAT Solver

The underlying SAT solver is invoked through subroutine $\mathsf {BoundedSAT}$, which is implemented using $\mathsf {CryptoMiniSat}$. Formally, $\mathsf {BoundedSAT}$ takes as inputs a formula *F*, a threshold $\mathsf {thresh}$, and a sampling set *S*, and returns a subset *Y* of $sol({F})_{\downarrow S}$, such that $|Y| = \min (\mathsf {thresh}, |sol({F})_{\downarrow S}|)$. The formula *F* consists of the original formula, which we want to count or sample, conjuncted with a set of XOR constraints defined through a hash function sampled from the family $\mathcal {H}_{xor}$. We henceforth denote such formulas as CNF-XOR formulas. Note that the efficient encoding of XOR constraints into CNF requires the introduction of new variables and hence the sampling set *S* usually does not contain all variables in *F*.

As is consistent with prior studies, profiling of $\mathsf {ApproxMC3}$ and $\mathsf {UniGen2}$ reveal that over 99% of the time is spent in the runtime of $\mathsf {BoundedSAT}$. Therefore recent efforts have focused on improving $\mathsf {BoundedSAT}$. Soos and Meel [19] sought to address the performance of the underlying SAT solver by proposing a new architecture, called $\mathsf {BIRD}$, that allows the usage of in- and pre-processing techniques for a Gauss Jordan Elimination (GJE)-augmented SAT solver. $\mathsf {ApproxMC2}$, integrated with $\mathsf {BIRD}$, called $\mathsf {ApproxMC3}$, gave up to three orders of magnitude runtime performance improvement. Such significant improvements are rare in the SAT community. Encouraged by Soos and Meel’s observations, we seek to build on top of $\mathsf {BIRD}$ to achieve an even tighter integration of the underlying SAT solver and $\mathsf {ApproxMC3}$/$\mathsf {UniGen2}$.

$\varvec{\mathsf {BIRD}}$**: Blast, Inprocess, Recover, and Destroy.** Pre- and inprocessing techniques are known to have a large impact on the runtime performance of SAT solvers. However, earlier Guassian elimination architectures were unable to perform these techniques. Motivated by this inability, Soos and Meel [19] proposed a new framework, called $\mathsf {BIRD}$, that allows usage of inprocessing techniques for GJE-augmented CDCL solvers. The key idea of $\mathsf {BIRD}$ is to blast XOR clauses into CNF clauses so that any technique working solely on CNF clauses does not violate soundness of the solver. To perform Gauss-Jordan elimination, one needs efficient algorithms and data structures to extract XORs from CNF. The entire framework is presented as follows: 
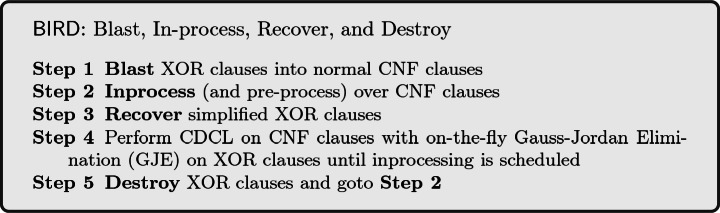
 The above loop terminates as soon as a satisfying assignment is found or the formula is proven $\mathsf {UNSAT}$. The $\mathsf {BIRD}$ architecture separates inprocessing from CDCL solving and therefore every sound inprocessing step can be employed.

## Technical Contributions to CNF-XOR Solving

Inspired by the success of $\mathsf {BIRD}$, we seek to further improve the underlying SAT solver’s architecture based on the queries generated by the hashing-based techniques. To this end, we relied on extensive profiling of $\mathsf {CryptoMiniSat}$ augmented with $\mathsf {BIRD}$ to identify the key performance bottlenecks, and propose solutions to overcome some of the challenges.

### Detaching XOR Clauses from Watch-Lists

Given a formula *F* in CNF, the recovery phase of $\mathsf {BIRD}$ attempts to construct a set of XORs, *H* such that $F \rightarrow H$. As detailed in [19], the core technique for recovery of an XOR of size *k* is to establish whether the required $2^{k-1}$ combinations of clauses are implied by the existing CNF clauses. For example, the XOR $x_1 \oplus x_2 \oplus x_3 = 0$ (where $k = 3$) can be recovered if the existing set of CNF clauses implies the following $4 (= 2^{3-1} )$ clauses: $(x_1 \vee x_2 \vee \lnot x_3) \wedge (x_1 \vee \lnot x_2 \vee x_3) \wedge (\lnot x_1 \vee x_2 \vee x_3) \wedge (\lnot x_1 \vee \lnot x_2 \vee \lnot x_3)$. To this end, the first stage of the recovery phase of $\mathsf {BIRD}$ iterates over the CNF clauses and for a given clause, called $\mathsf {base\_cl}$ of size *k*, searches whether the remaining $2^{k-1}-1$ clauses are implied as well, in which case the resulting XOR is added. It is worth noting that a clause can imply multiple clauses over the the set of variables of $\mathsf {base\_cl}$; For example if the $\mathsf {base\_cl}$ = $(x_1 \vee \lnot x_2 \vee x_3)$, then the clause $(\lnot x_1)$ would imply the two clauses $(\lnot x_1 \vee \lnot x_2 \vee \lnot x_3 )$ and $(\lnot x_1 \vee x_2 \vee x_3)$. Note that given a $\mathsf {base\_cl}$, we are only interested in clauses over the variables in $\mathsf {base\_cl}$.

During blasting of XORs into CNF, XORs are first cut into smaller XORs by introducing auxiliary variables. Hence, the first stage of recovery phase must recover these smaller XORs and the second phase reconstructs the larger XORs by XOR-ing two XORs together if they differ only on one variable, referred to as a *clash variable*. For example, $x_1 \oplus x_2 \oplus x_3 = 0$ and $x_3 \oplus x_4 \oplus x_5 = 1$ can be XOR-ed together over clash variable $x_3$ to obtain $x_1 \oplus x_2 \oplus x_4 \oplus x_5 = 1$.

Since $\mathsf {BIRD}$ performs CDCL in tandem with Gauss-Jordan elimination, it is worth noting that the Gauss-Jordan elimination (GJE)-based decision procedure is sound and complete, i.e., all unit propagations and conflicts implied by the given set of XORs would be discovered by a GJE-based decision procedure. For the initial formula (in CNF) *F* and the recovered set of XORs, *H*, if a set of CNF clauses *G* is implied by *H*, then presence or absence of *G* does not affect soundness and completeness of GJE-augmented CDCL engine. Our extensive profiling of the $\mathsf {BIRD}$ framework integrated in $\mathsf {CryptoMiniSat}$ revealed a significant time spent in examination of clauses in *G* during unit propagation. To this end, we sought to ask how to design an efficient technique to find all the CNF clauses implied by the recovered XORs. These clauses could be detached from unit propagation without any negative effect on correctness of execution.

A straightforward approach would be to mark all the clauses during the blasting phase of XORs into CNF. However, the incompleteness of the recovery phase of $\mathsf {BIRD}$ does not guarantee that all such marked clauses are indeed implied by the recovered set of XORs. Another challenge in the search for detachable clauses arises due to construction of larger XORs by combining smaller XORs. For example, while $x_1 \oplus x_2 \oplus x_3 = 0$ and $x_3 \oplus x_4 \oplus x_5 = 1$ imply $(x_1 \vee x_2 \vee \lnot x_3)$ and $(x_3 \vee x_4 \vee x_5)$, the combined XOR $x_1 \oplus x_2 \oplus x_4 \oplus x_5 = 1$ does not imply $(x_1 \vee x_2 \vee \lnot x_3)$ and $(x_3 \vee x_4 \vee x_5)$.

Two core insights inform our design of the modification of the recovery phase and search for detachable clauses. Firstly, given a base clause $\mathsf {base\_cl}$, if a clause $\mathsf {cl}$ participates in the recovery of XORs over the variables in $\mathsf {base\_cl}$, then $\mathsf {cl}$ is implied by the recovered XOR if the number of variables in $\mathsf {cl}$ is the same as that of $\mathsf {base\_cl}$. We call such a clause $\mathsf {cl}$ a *fully participating clause*. Secondly, let $G_1$ and $G_2$ be the set of CNF clauses implied by two XORs $q_1$ and $q_2$ that share exactly one variable, say $x_i$. Let $U = (\mathsf {Vars }(q_1) \cup \mathsf {Vars }(q_2))$ $\setminus$ $x_i$. Let $q_3$ be the XOR obtained by XORing together $q_1$ and $q_2$, then, $sol({q_3})_{\downarrow U} \subseteq sol({G_1 \wedge G_2})_{\downarrow U}$ if $x_i$ does not appear in the remaining clauses, i.e., $x_i \notin \mathsf {Var}\left[ {F \setminus (G_1 \cup G_2)}\right]$.

The above two insights lead us to design a modified recovery and detachment phase as follows. During recovery, we add every *fully participating clause* to the set of detachable clauses *D*. Let $$\mathcal {U} = S \cup (\mathsf {Vars }(D) \cap \mathsf {Vars }(F$$ $$\setminus $$ $$D))$$. Then, the recovery of longer XORs is only performed over clash variables that do not belong to $$\mathcal {U}$$. We then detach the clauses in *D* from watch-lists during GJE-augmented CDCL phase, mark the clash variables as non-decision variables, perform CDCL, and only reattach the clauses and re-set the clash variables to be decision variables after the Destroy phase of $$\mathsf {BIRD}$$.

If the formula is satisfiable, the design of the solver is such that the solution is always found during the GJE-augmented CDCL solving phase. Since clauses in *D* are detached and the clash variables are set to be not decided on during this phase, the clash variables are always left unassigned. As discussed below, however, we only need to extract solutions over the sampling set *S*, therefore the solution found is adequate as-is, without the clash variables, which are by definition not over *S* as they are only introduced for having short encodings of XORs into CNF.

Conceptually, this approach reconciles the overhead introduced by $\mathsf {BIRD}$, i.e., that XOR constraints are also present as regular clauses, with the neatness of the original $\mathsf {CryptoMiniSat}$ that stored XOR and regular constraints in different data structures. This reconciliation takes the best of both worlds.

### Fast Propagation/Conflict Detection and Reason Generation

We identified two key bottlenecks in the the current GJE component of $\mathsf {BIRD}$ framework integrated in $\mathsf {CryptoMiniSat}$, which we sought to improve upon. To put our contributions in the context, we first describe the technical details of the core data structures and algorithms.

**Han-Jiang’s GJE.** To perform Gaussian elimination on a set of XORs, the XORs are represented as a matrix where each row represents an XOR and each column represents a variable. The framework proposed by Soos et al. updates the matrix whenever a variable is assigned and removes the assigned variable from all XORs by zeroing out the corresponding column. However, using the matrix in such a way involves significant memory copying during backtracking due to having to revert the matrix to a previous version.

To avoid the overhead, Han and Jiang proposed a new framework [13] building on Simplex-like techniques that performs Gauss-Jordan elimination, i.e., using reduced row echelon form instead of row echelon form. The key data structure innovation was to employ a two-watched variable scheme for each row of the matrix wherein the watched variables are called basic and non-basic variables. Essentially, the basic variables are the variables on the diagonal of a matrix in reduced row echelon form and hence every row has exactly one basic variable and the basic variable only occurs in one row. Similar to standard CDCL solving, when a matrix row’s watch is assigned, the GJE component must determine whether the row (1) propagates, (2) needs to assign a new watch, (3) is satisfied, or (4) is conflicted. It is worth recalling that a row would propagate if all except one variable has been assigned and would conflict or be satisfied if all the variables in a row have been assigned. Furthermore, we need to find a new watch if a watched variable was assigned and there is more than one unassigned variable left. If a basic variable is replaced by a new watch then the two corresponding columns are swapped and the reduced row echelon form is recomputed. In practice swapping columns is avoided by keeping track of which column is a basic variable.

For propagation, checking for conflict, and conflict clause generation Han-Jiang proposed a sequential walk through a row that eagerly computes the reason clause and stops when it encounters a new watch variable or reaches until the end of the row. At that point, the system (1) knows whether the row is satisfied, propagating, or conflicted, and (2) if not satisfied, has eagerly computed the reason clause for the propagation or the conflict.

For general benchmarks where XOR constraints do not play an influential role in determining satisfiability of the underlying problem, the GJE component can be as small as 10% of the entire solving time. However, for formulas generated generated by hashing-based techniques, our profiling demonstrated several cases where the Gaussian elimination component could be very time consuming, taking up to 90% of solving time.

While the choice of GJE combined with clever data structure maintenance led to significant improvements of the runtime of Gaussian Elimination component, our profiling identified two processes as key bottlenecks: propagation checking and reason generation. We next discuss our proposed algorithmic improvements that achieve significant runtime improvement by addressing these bottlenecks.

**Tinted Fast Unit Propagation.** The core idea to achieve faster propagation is based on bit-level parallelism via the different native operations supported by modern CPUs. In particular, modern CPUs provide native support for basic bitwise operations on bit fields such as AND, INVERT, hamming weight computation (i.e., the number of non-zero entries), and *find first set* (i.e., finding the index of first non-zero bit). Given the widespread support of SIMD extensions, the above operations can be performed at the rate of 128...512 bits per instruction. Therefore, the core data structure represents every 0-1 vector as a bit field.

A set of XORs over *n* variables $x_1,\dots ,x_n$ is represented as $\textit{\textbf{ M }}\textit{\textbf{ x }} = \textit{\textbf{ b }}$ for a 0-1 matrix $\textit{\textbf{ M }}$ of size $m \times n$, 0-1 vector *b* of length *m* and $\textit{\textbf{ x }} = (x_1, \dots , x_n)^T$. Consider the $i-$th row of $\textit{\textbf{ M }}$, denoted by $\textit{\textbf{ M }}[i]$. Let $\textit{\textbf{ a }}$ be a 0-1 vector of size *n* such that $\textit{\textbf{ a }}[j]$=1 if the variable $x_j$ is assigned True or False, and 0 in case $x_j$ is unassigned. Let $\textit{\textbf{ v }}$ be a 0-1 vector of size *n* such that $\textit{\textbf{ v }}[j] = 1$ if $x_j$ is set to True and 0 otherwise. Let $\overline{\textit{\textbf{ z }}}$ be the bitwise inverse of a 0-1 vector $\textit{\textbf{ z }}$ and & be the bitwise AND operation. Let $W_{unass}=\textsf {hamming\_weight}( \overline{\textit{\textbf{ a }}}  \&  \textit{\textbf{ M }}[i] )$ the number of unassigned variables in the XOR represented by row *i*, and $W_{val}=\textsf {hamming\_weight}(\textit{\textbf{ v }}  \&  \textit{\textbf{ M }}[i])$ the number of satisfied variables. We view the computation of $W_{unass}$ and $W_{val}$ as viewing the world of $\textit{\textbf{ M }}$ through the tinted lens of $\textit{\textbf{ v }}$ and ${\overline{\textit{\textbf{ a }}}}$. Now, the following holds:

1. labelrow:satisifed Row *i* is satisfied if and only if $W_{unass}=0$ and $(W_{val} \mod 2) \oplus \textit{\textbf{ b }}[i] = 0$.
2. Row *i* causes a conflict if and only if $W_{unass}=0$ and $(W_{val} \mod 2) \oplus \textit{\textbf{ b }}[i] = 1$.
3. Row *i* propagates if and only if $W_{unass}=1$. Propagated variable is the one that corresponds to the column with the only bit set in $\overline{\textit{\textbf{ a }}} \&  M[i]$. The value propagated is $(W_{val} \mod 2) \oplus \textit{\textbf{ b }}[i]$.
4. A new watch needs to be found for row *i* if and only if $W_{unass}\ge 2$. The new watch is any one of the variables corresponding to columns with the bits set to 1 in $\overline{\textit{\textbf{ a }}} \&  \textit{\textbf{ M }}[i]$, except for the already existing watch variable.

*Reason Generation.* For propagation and conflict we generate the reason clauses for row *i* as follows. We forward-scan $\textit{\textbf{ M }}[i]$ for all set bits and insert the corresponding variable into the reason clause as a literal that evaluates to false under the current assignment. In the case of propagation, the literal added for the propagated variable, say $x_j$, is added as literal $\lnot x_j$ if $(W_{val}\mod 2)\oplus \textit{\textbf{ b }}[i]=0$ and $x_j$ otherwise.

*Example.* For example, let $\textit{\textbf{ b }}[i]=1$ and $\textit{\textbf{ M }}[i]=10011$ corresponding to variables $x_1, x_2, \ldots x_5$ and assignments 1?11? respectively, where “?” indicates an unassigned variable. Then $\textit{\textbf{ a }}=10110, \overline{\textit{\textbf{ a }}}  \&  \textit{\textbf{ M }}[i]=00001, W_{unass}=1, \textit{\textbf{ v }}=10110, \textit{\textbf{ v }} \&  \textit{\textbf{ M }}[i]=10010, W_{val}=2$ and $(W_{val}\mod 2)\oplus \textit{\textbf{ b }}[i]=1$. Therefore, this row propagates (case 3 above), and the reason generated is $(\lnot x_1 \vee \lnot x_{4} \vee x_{5})$. If the assignements were 11110, then $W_{unass}=0$ and $(W_{val}\mod 2)\oplus \textit{\textbf{ b }}[i]=1$ so this row conflicts (case 2 above), with conflict clause $(\lnot x_{1} \vee \lnot x_{4} \vee x_{5})$.

**Performance.** Notice that all cases only require bitwise and, inverse, hamming weight and find first set operations. To find a new watch in case 4 we first find the first bit that is set to 1 in $\bar{\textit{\textbf{ a }}}  \&  \textit{\textbf{ M }}$ by invoking find first set. In case the obtained index is the same as the existing watch variable, we remove the first 1-bit by left shifting and run find first set again to find the second 1-bit. Bitwise and and inverse are trivially single-assembly instructions. We use compiler intrinsics to execute find first set and hamming weight functions, which compile down to BSF and POPCNT in x86 assembly, respectively. It is worth pointing out that we keep the bit field representations of $\textit{\textbf{ a }}$ and $\textit{\textbf{ v }}$ synchronized when variables are assigned. During backtracking we reset these to zero and refill them as needed. For better cache efficiency, we use sequential set of bit-packed 64-bit integers to represent all bit-fields, rows, and matrices.

Although bit-packing is not a novel concept in the context of CNF-XOR solving, let us elaborate why we believe that our contribution is conceptually interesting. Soos et al. [20] used bit-packed pre- and post-evaluated matrices. Since post-evaluated matrices lose information, they have to be saved and reloaded on backtracking. Han and Jiang’s code [13] changed this to using pre-evaluated matrices only, which free the system from having to save and reload. But it was slow, because bit-by-bit evaluation had to happen on every matrix row read (thanks to the missing post-evaluation matrix). Our improved approach is essentially merging the best of both worlds: fast evaluation, without having to save and reload.

### Lazy Reason Clause Generation

As discussed earlier, the current $\mathsf {BIRD}$ performs eager reason clause generation in a spirit similar to the original proposal by Han and Jiang. At the time of proposal of eager clause generation by Han and Jiang, the state of the art SAT solver at that time could solve problems with XOR clauses of sizes in few tens to few hundreds. The improved scalability, however, highlights the overhead due to eager reason clause generation. During our profiling, we observed that for several problems, the independent support of the underlying formula ranges in thousands, and therefore, leading to generation of reason clauses involving thousands of variables. The generation of such long reason clauses is time consuming and tedious. Furthermore, a significant fraction of reason clauses are never required during conflict analysis phase as we are, often, focused only on finding a 1UIP clause. Therefore, we seek to explore lazy reason clause generation.

Let the state of a clause *c* indicate whether *c* is satisfied, conflicted or undetermined (i.e., the clause is neither satisfied nor conflicted). The core design of our lazy generation technique is based on the following invariant satisfied by CDCL-based techniques: Once a (CNF/XOR) clause is satisfied or conflicted, the assignment to the variables in the clause does not change as long the state of the clause does not change. Observe that when a clause propagates, the propagated literal changes the state of the clause to satisfied. Furthermore, as long as all variables are assigned, the row will not participate in GJE because none of the contained variables can become a basic watch. Therefore, whenever an XOR clause propagates, we keep an index of the row and the propagating literal but do not compute the reason clause. Now, whenever a reason clause is requested, we compute the reason clause as detailed above and return a pointer to the computed reason clause, and index the computed clause by the corresponding row. To ensure correctness, whenever a row causes a propagation, we delete the existing reason clause but we do not eagerly compute the new corresponding reason clause. On the other hand, if a row is conflicting, the conflict analysis requires the reason clause immediately and as such the reason clause is eagerly computed.

Lazy reason clause generation allows us to skip the majority of reason clauses to be generated. Furthermore, given that a row cannot lead to more than one reason clause, it allows us to statically allocate memory for them. This is in stark contrast to the original implementation that not only eagerly computed all reason clauses, but also dynamically allocated memory for them, freeing the memory up during backtracking.

### Skipping Solution Extension of Eliminated Variables

SAT solvers aim to present a clean and uncomplicated API interface with internal behavior typically hidden to enable fast pacing development of heuristics without necessitating change in the interface for the end users. While such a design philosophy allows easier integration, it may be an hindrance to achieving efficiency for the use cases that may not be seeking a simple off-the-shelf behavior. Given the surge of projected counting and sampling as the desired formulation, $\mathsf {BoundedSAT}$ is invoked with a sampling set and we are interested only in the assignment to variables in the sampling set. A naive solution would be to obtain a complete assignment over the entire set of variables and then extract an assignment over the desired sampling set. In this context, we wonder if we can terminate early after the variables in the sampling set are assigned. In modern SAT solvers, once the solver has determined that the formula is satisfied, the *solution extension* subroutine is invoked that extends the current partial assignment to a complete assignment. Upon profiling, we observed that, during solution extension, a significant time is spent in computing an assignment to the variables eliminated due to Bounded Variable Elimination (BVE) [12] during pre- and inprocessing. When a solution is found, the eliminated clauses must be re-examined in reverse, linear, order to make sure the eliminated variables in the model are correctly assigned. This examination process can be time-consuming on large instances with large portions of the CNF eliminated.

BVE is widely used in modern SAT solvers owing to its ability to eliminate a large subset of the input formula and thereby allowing compact data structures. While disabling BVE would eliminate the overhead during solution extension phase, it would also significantly degrade performance during solving phase. Since we are interested in solutions only over the sampling set, we disable the invocation of bounded variable elimination for variables in the sampling set. Therefore, whenever the SAT solver determines that the current partial assignment satisfies the formula, all the variables in the sampling set are assigned and we do not invoke solution extension. The disabling of solution extension can save significant (over 20%) time on certain instances.

### Putting It All Together: $\mathsf {BIRD2}$

We combine improvements proposed above into our new framework, called $\mathsf {BIRD2}$, a namesake to capture the primary architecture of Blast, In-process, Recover, Detach, and Destroy. For completeness, we present the core skeleton of $\mathsf {BIRD2}$ in Algorithm 1. $\mathsf {BIRD2}$ terminates as soon as a satisfying assignment is found or the formula is proven $\mathsf {UNSAT}$. Similar to $\mathsf {BIRD}$, $\mathsf {BIRD2}$ architecture separates inprocessing from CDCL solving and therefore every sound inprocessing step can be employed.
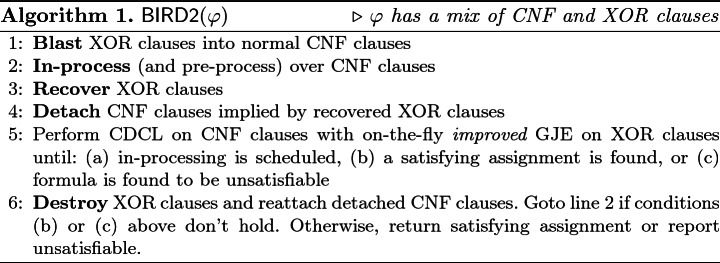


## Technical Contribution to Counting and Sampling

In this section, we discuss our primary technical contribution to hashing-based sampling and counting techniques.

### Reuse of Previously Found Solutions

The usage of a prefix-slicing ensures monotonicity of the random variable, $\mathsf {Cnt}_{\langle F, i \rangle }$, since from the definition of prefix-slicing, we have that for all *i*, $h^{(i+1)}(x) = \alpha ^{(i+1)} \implies h^{(i)}(x) = \alpha ^{(i)}$. Formally,

#### Proposition 1

For all $$1\le i <m$$, $$\mathsf {Cell}_{\langle F, i+1 \rangle } \subseteq \mathsf {Cell}_{\langle F, i \rangle }$$

Furthermore as is evident from the analysis of $\mathsf {ApproxMC3}$ [10], the pairwise independence of the family $\mathcal {H}_{xor}$ implies $\frac{\mathsf {E}[\mathsf {Cnt}_{\langle F, i \rangle }]}{\mathsf {E}[\mathsf {Cnt}_{\langle F, j \rangle }]} = 2^{j-i}$. Therefore, once we obtain the set of solutions from invocation of $\mathsf {BoundedSAT}$ for $F \wedge (h^{i})^{-1}(\mathbf {0})$ (i.e., after putting *i* XORs), we can potentially reuse the returned solutions when we are interested in enumerating solutions for $F \wedge (h^{j})^{-1}(\mathbf {0})$. In particular, note that if $i > j$, then Proposition 1 implies that all the solutions $F \wedge (h^{i})^{-1}(\mathbf {0})$ are indeed solutions for $F \wedge (h^{j})^{-1}(\mathbf {0})$ and we can invoke $\mathsf {BoundedSAT}$ with adjusted threshold. On the other hand, for $i < j$, we can check if the solutions of $F \wedge (h^{i})^{-1}(\mathbf {0})$ also satisfy $F \wedge (h^{i+1})^{-1}(\mathbf {0})$.

On closer observation, we find that the latter case may not be always helpful when *i* and *j* differ by more than a small constant since the ratio of their expected number of solutions decreases exponentially with $j-i$. Interestingly, as discussed in Sect. 3, both $\mathsf {ApproxMC3}$ and $\mathsf {UniGen2}$ employ linear search over intervals of sizes 4 to 7. for the right values of *m*. In particular, for both $\mathsf {ApproxMC3}$ and $\mathsf {UniGen2}$, the linear search seeks to identify a value of $m^*$ such that $\mathsf {Cnt}_{\langle F, m^*-1 \rangle } \ge \mathsf {thresh}$ and $\mathsf {Cnt}_{\langle F, m^* \rangle } < \mathsf {thresh}$ for an appropriately chosen $\mathsf {thresh}$. Therefore, when invoking $\mathsf {BoundedSAT}$ for $i=k$ after determining that for $i=k+1$, ${\mathsf {Cnt}_{\langle F, k+1 \rangle }} < \mathsf {thresh}$, we can replace $\mathsf {thresh}$ with $\mathsf {thresh}- \mathsf {Cnt}_{\langle F, k+1 \rangle }$. Similarly, when invoking $\mathsf {BoundedSAT}$ for $i=k$ after determining that for $i=k-1$, $\mathsf {Cnt}_{\langle F, k-1 \rangle } \ge \mathsf {thresh}$, we first check how many solutions of $F \wedge (h^{k-1})^{-1}(\mathbf {0})$ satisfy $F \wedge (h^{k})^{-1}(\mathbf {0})$. As noted above, in expectation, $\mathsf {thresh}/2$ out of $\mathsf {thresh}$ solutions of $F \wedge (h^{k-1})^{-1}(\mathbf {0})$ would satisfy $F \wedge (h^{k})^{-1}(\mathbf {0})$.

### $\mathsf {ApproxMC4}$ and $\mathsf {UniGen3}$

That said, we turn our focus to hashing-based sampling and counting techniques to showcase the impact of $\mathsf {BIRD2}$. To this end, we integrate $\mathsf {BIRD2}$ along with the proposed technique in Sect. 5.1 into the state of the art hashing-based counting and sampling tools: $\mathsf {ApproxMC3}$ and $\mathsf {UniGen2}$ respectively. We call our improved counting tool $\mathsf {ApproxMC4}$ and our improved sampling tool $\mathsf {UniGen3}$.

**Assurance of Correctness.** We believe it to be imperative to strongly verify correctness and quality of results provided by our tools, as it is not only possible but indeed easy to accidentally generate incorrect or low quality results, as demonstrated by Chakraborty and Meel [5]. To ensure the quality and correctness of our sampler and counter, we used three methods: (1) fuzzed the system as first demonstrated in SAT by Brummayer et al. [3], (2) compared the approximate counts returned by $\mathsf {ApproxMC4}$ with the counts computed by a known good exact model counter as previously performed by Soos and Meel [19], and (3) compared the distribution of samples generated by UniGen4 on an example problem against that of a known good uniform sampler as previously performed by Chakraborty et al. [9]. We focus on (1), i.e. fuzzing, here and defer the discussion about (2) and (3) to the next section.

Fuzzing is a technique [17] used to find bugs in code by generating random inputs and observing crashes, invariant check fails, and other errors from the output of the system under test. $\mathsf {CryptoMiniSat}$ has such a built-in fuzzer generating random CNFs and verifying the output of the solver. To account for XOR constraints, we improved the built-in fuzzer of $\mathsf {CryptoMiniSat}$ by adding a counting- and sampling-specific XOR-CNF generator. This inserts randomly generated XORs that form distinct matrices inside the generated CNFs and adds a randomly generated sampling set over some of these matrices. We also added hundreds of lines of invariant checks to our improved Gauss-Jordan elimination algorithm, running throughout our fuzzing tests. Running this improved fuzzer for many hundreds of CPU hours has greatly helped debugging and gaining confidence in our implementation.

## Evaluation

To evaluate the performance and quality of approximations and samples computed by $\mathsf {ApproxMC4}$ and $\mathsf {UniGen3}$, we conducted a comprehensive study involving 1896 benchmarks as released by Soos and Meel [16] comprising a wide range of application areas including probabilistic reasoning, plan recognition, DQMR networks, ISCAS89 combinatorial circuits, quantified information flow, program synthesis, functional synthesis, logistics, and the like.

In the context of counting, we focused on a comparison of the performance of $\mathsf {ApproxMC4}$ vis-a-vis $\mathsf {ApproxMC3}$. In the context of sampling, a simple methodology would have been a comparison of $\mathsf {UniGen3}$ vis-a-vis the state of the art sampler, $\mathsf {UniGen2}$. Such a comparison, in our view, would be unfair to $\mathsf {UniGen2}$ as while $\mathsf {ApproxMC3}$ builds on $\mathsf {BIRD}$ framework, such is not the case for $\mathsf {UniGen2}$. It is worth noting that the $\mathsf {BIRD}$ framework, proposed by Soos and Meel [19], can work as a drop-in replacement for the SAT solver in $\mathsf {UniGen2}$, as it only changes the underlying SAT solver. Therefore, we used $\mathsf {UniGen2}$ augmented with $\mathsf {BIRD}$, called $\mathsf {UniGen2}$+$\mathsf {BIRD}$ henceforth, as baseline for performance comparisons in the rest of this paper, as it is significantly faster than UniGen2, and therefore, will lead to a fair comparison and showcase improvements solely due to $\mathsf {BIRD2}$.

To keep in line with prior studies, we set $\varepsilon = 0.8$ and $\delta = 0.8$ for $\mathsf {ApproxMC3}$ and $\mathsf {ApproxMC4}$ respectively. Similarly, we set $\varepsilon = 16$ for both $\mathsf {UniGen3}$ and $\mathsf {UniGen2}$+$\mathsf {BIRD}$ respectively. The experiments were conducted on a high performance computer cluster, each node consisting of 2xE5-2690v3 CPUs with 2 $\times$ 12 real cores and 96 GB of RAM. We use a timeout of 5000 s for each experiment, which consisted of running a tool on a particular benchmark.

### Performance

**Fig. 1.**
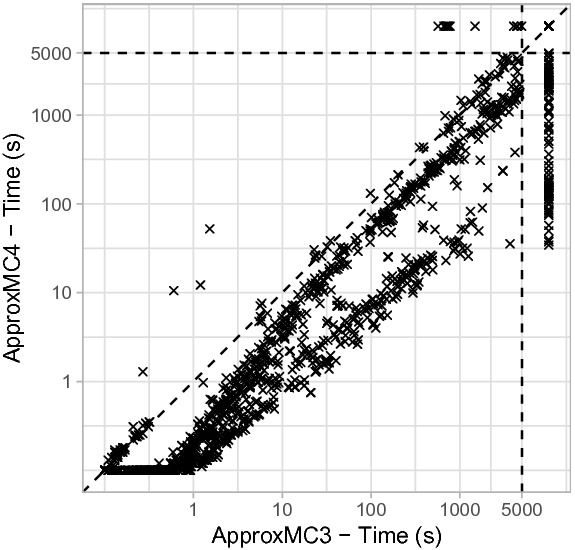
Comparison of $\mathsf {ApproxMC4}$ and $\mathsf {ApproxMC3}$. $\mathsf {ApproxMC4}$ is faster below the diagonal. Time outs are plotted behind the 5000 s mark.

$\varvec{\mathsf {ApproxMC4}}$ **vis-a-vis** $\varvec{\mathsf {ApproxMC3}}$. Figure 1 shows a scatter plot comparing $\mathsf {ApproxMC4}$ and $\mathsf {ApproxMC3}$. Although, there are some benchmarks that are solved faster with $\mathsf {ApproxMC3}$ there is a clear trend demonstrating the speed up achieved through our improvements: $\mathsf {ApproxMC4}$ can solve many benchmarks more than 10 times faster and in total solves 77 more instances than $\mathsf {ApproxMC3}$. In particular, $\mathsf {ApproxMC3}$ and $\mathsf {ApproxMC4}$ solved 1148 and 1225 instances respectively, while achieving PAR-2 scores of 4146 and 3701 respectively.

**Fig. 2.**
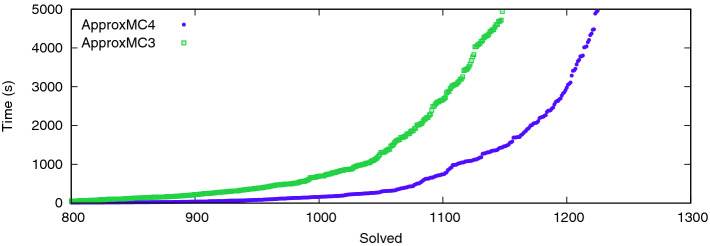
Cactus plot showing behavior of $\mathsf {ApproxMC4}$ and $\mathsf {ApproxMC3}$

Figure 2 shows the cactus plot for $\mathsf {ApproxMC3}$ and $\mathsf {ApproxMC4}$. We present the number of benchmarks on the x-axis and the time taken on the y-axis. A point (*x*, *y*) implies that *x* benchmarks took less than or equal to *y* seconds to solve for the corresponding tool.

To present a detailed picture of performance gain achieved by $\mathsf {ApproxMC4}$ over $\mathsf {ApproxMC3}$, we present a runtime comparison of $\mathsf {ApproxMC4}$ vis-a-vis $\mathsf {ApproxMC3}$ in Table 1 on a subset of benchmarks. Column 1 of the table presents benchmarks names, while columns 2 and 3 list the number of variables and clauses. Column 4 and 5 list the runtime (in seconds) of $\mathsf {ApproxMC4}$ and $\mathsf {ApproxMC3}$, respectively.

While investigating the large improvements in performance, we observed that when both the sampling set and the number of solutions is large for a problem, the new system can be up to an order of magnitude faster. In these cases the Gauss-Jordan elimination (GJE) component of the SAT solver dominated the runtime of $\mathsf {ApproxMC3}$ due to the large matrices involved in such problems. The improvements of $\mathsf {BIRD2}$ has led to significant improvement in efficiency of GJE component and we observe that the runtime, in such instance, is now often dominated by the CDCL solver’s propagation and conflict clause generation routines.

**Fig. 3.**
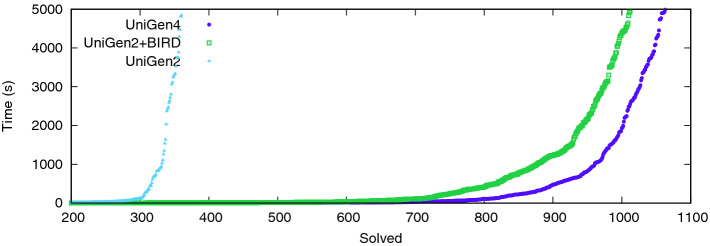
Sampling performance of $\mathsf {UniGen2}$ and $\mathsf {UniGen2}$+$\mathsf {BIRD}$ versus $\mathsf {UniGen3}$.

$\varvec{\mathsf {UniGen3}}$ **vis-a-vis** $\varvec{\mathsf {UniGen2}}$+$\varvec{\mathsf {BIRD}}$**.** Similar to Fig. 2, Fig. 3 shows the cactus plot for $\mathsf {UniGen3}$, $\mathsf {UniGen2}$+$\mathsf {BIRD}$, and $\mathsf {UniGen2}$. We present the number of benchmarks on the x-axis and the time taken on the y-axis. $\mathsf {UniGen3}$ and $\mathsf {UniGen2}$+$\mathsf {BIRD}$ were able to solve 1012 and 1063 instances, respectively while achieving PAR-2 scores of 4574 and 4878, respectively. $\mathsf {UniGen2}$ could solve only 360 benchmarks, thereby justifying our choice of implementing $\mathsf {UniGen2}$+$\mathsf {BIRD}$ as a baseline for fair comparison to showcase strengths of $\mathsf {BIRD2}$. We would like to highlight that the cactus plot shows that given a 2600 s timeout, $\mathsf {UniGen}$ can sample as many benchmarks as $\mathsf {UniGen2}$+$\mathsf {BIRD}$ would do for a 5000 s timeout.

**Table 1.** Performance comparison of $\mathsf {ApproxMC3}$ vis-a-vis $\mathsf {ApproxMC4}$ and $\mathsf {UniGen2}$+$\mathsf {BIRD}$ vis-a-vis $\mathsf {UniGen3}$. TO indicates timeout after 5000 s or out of memory. Notice that on many problems that used to time out even for counting, we can now confidently sample.

Benchmark	Vars	Cls	$\mathsf {ApproxMC3}$	$\mathsf {ApproxMC4}$	$\mathsf {UniGen2}$+$\mathsf {BIRD}$	$\mathsf {UniGen3}$
			time (s)	time (s)	500 samples time (s)	500 samples time (s)
or-70-5-1-UC-20	140	350	6.03	2.07	14.21	6.08
prod-4	7497	37358	56.65	7.09	171.57	36.54
min-8	1545	4230	152.53	5.58	471.47	35.04
parity.sk_11_11	13116	47506	389.26	436.32	705.85	809
leader_sync4_11	205198	129149	346.4	20.55	1019.09	106.93
blasted_TR_b12_2	2426	8373	308.08	20.46	1218.01	546.62
hash-8-6	377545	1517574	462.28	266.59	1321.91	633.84
s15850a_15_7	10995	24836	1206.17	31.69	2782.96	230.17
ConcreteRole	395951	1520924	1694.19	309.07	3083.99	923.69
tire-3	577	2004	3059.19	233.28	3876.03	797.42
04B-2	19510	86961	1860.97	625.81	TO	2236.31
blasted_case138	849	2253	TO	3691.9	TO	TO
hash-11-4	518449	2082039	4602.95	4043.4	TO	TO
karatsuba.sk_7_41	19594	82417	3192.85	3410.36	TO	TO
log-3	1413	29487	TO	123.15	TO	408.25
modexp8-8-6	167793	633614	4439.21	TO	TO	TO
or-100-5-6-UC-20	200	500	TO	1689.47	TO	4898.43
prod-28	52233	261422	TO	235.02	TO	1053.9
s38417_15_7	25615	57946	TO	187.71	TO	TO
signedAvg	30335	91854	TO	114.15	TO	582.01

To present a clear picture of performance gain by $\mathsf {UniGen3}$ over $\mathsf {UniGen2}$+$\mathsf {BIRD}$, we present runtime comparison for $\mathsf {UniGen3}$ vis-a-vis $\mathsf {UniGen2}$+$\mathsf {BIRD}$ in Table 1, where in addition to data on $\mathsf {ApproxMC3}$ and $\mathsf {ApproxMC4}$, columns 5 and 6 lists the runtime for $\mathsf {UniGen3}$ and $\mathsf {UniGen2}$+$\mathsf {BIRD}$ respectively. Similar to the observation above, we note that $\mathsf {UniGen3}$ is able to sample for instances that timed out even for $\mathsf {ApproxMC3}$. It is worth to recall that $\mathsf {UniGen3}$ (and $\mathsf {UniGen2}$) first makes a call to an approximate counter during its parameter search phase.

#### Remark 1

Since the runtime improvements of $\mathsf {ApproxMC4}$ and $\mathsf {UniGen3}$ are primarily due to improvements in the underlying SAT solver, it is worth pointing out, to put our contribution in context, that the difference between average PAR-2 scores of the top two solvers in a SAT competition is usually less than 100.

**Fig. 4.**
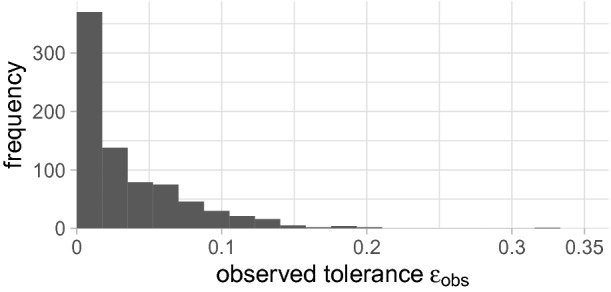
The histogram of the observed tolerance $\varepsilon _{obs}$ shows that the approximate counts are very close to the exact counts.

### Quality and Correctness

**Quality of Counting.** To evaluate the quality of approximation we follow the same approach as Soos and Meel [19] and compare the approximate counts returned by $\mathsf {ApproxMC4}$ with the counts computed by an exact model counter, namely DSharp^4^. The approximate counts and the exact counts are used to compute the observed tolerance $\varepsilon _{obs}$, which is defined as max($\frac{|sol({F})_{\downarrow S}|}{\mathrm {AprxCount}}-1,\frac{\mathrm {AprxCount}}{|sol({F})_{\downarrow S}|}-1$), where $\mathrm {AprxCount}$ is the estimate computed by $\mathsf {ApproxMC4}$ for a formula *F* and a sampling set *S*, which are both given for each benchmark. Note that, using $\varepsilon _{obs}$, we can rewrite the theoretical $(\varepsilon ,\delta )$-guarantee to $\mathsf {Pr}[\varepsilon _{obs} \le \varepsilon ] \ge 1 - \delta$ and hence we expect that $\varepsilon _{obs}$ is mostly below $\varepsilon = 0.8$. The observed tolerance $\varepsilon _{obs}$ over all benchmarks is shown in Fig. 4. We observe a maximal value for $\varepsilon _{obs}$ of 0.3333 and the the arithmetic mean of $\varepsilon _{obs}$ across all benchmarks is 0.0411. Hence, the approximate counts are much closer to the exact counts than is theoretically guaranteed.

**Fig. 5.**
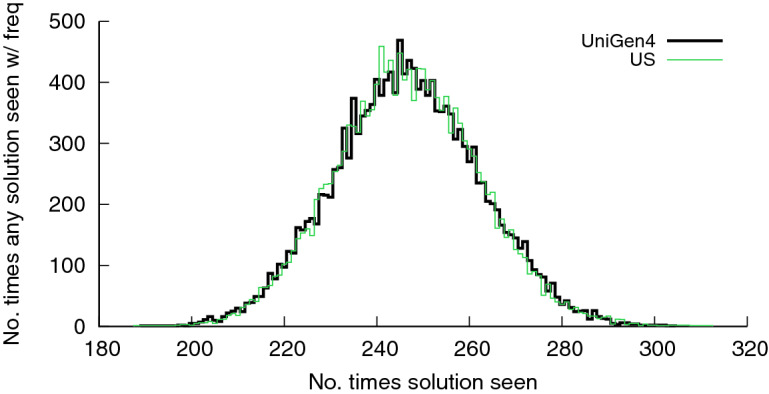
Distribution of solution recurrence as generated by $\mathsf {UniGen3}$ and $\mathsf {US}$ for the CNF blasted_case110.cnf.

**Quality of Sampling.** To evaluate the quality of sampling, we employed the uniformity tester, $\mathsf {Barbarik}$, proposed by Chakraborty and Meel [5]. To this end, we selected 35 benchmarks from the pool of benchmarks employed by Chakraborty and Meel in their work and we tested $\mathsf {UniGen3}$ for all the 35 benchmarks. We observed that $\mathsf {Barbarik}$ accepts $\mathsf {UniGen3}$ for all the 35 instances, thereby providing a certificate for uniformity. We refer the reader to [5] for detailed discussion of the guarantees provided by $\mathsf {Barbarik}$. Keeping in line with past work on sampling that tries to demonstrate the quality of sampling on a representative benchmark where exact uniform sampling is feasible via enumeration-based techniques, we chose the CNF instance *blasted_case110* (287 variables and 16384 solutions), which has been chosen in the previous studies as well. To this end, we implemented a simple ideal uniform sampler, denoted by $\mathsf {US}$ henceforth, by enumerating all the solutions and then picking a solution uniformly at random. We then generate 4, 039, 266 samples from both $\mathsf {UniGen3}$ and $\mathsf {US}$. In each case, the number of times various witnesses were generated was recorded, yielding a distribution of the counts. Fig. 5 shows the distributions of counts generated versus # of solutions. The *x*-axis represents counts and the *y*-axis represents the number of witnesses appearing the specified number of times. Thus, the point (230,212) represents the fact that each of 212 distinct witnesses were generated 230 times among the 4, 039, 266 samples. While $\mathsf {UniGen3}$ provides guarantees of almost-uniformity only, the two distributions are statistically indistinguishable. In particular, the KL divergence [15] of the distribution by UniGen from that of $\mathsf {US}$ is 0.003989.

## Conclusions

We investigated the bottlenecks of CNF-XOR solving in the context of hashing-based approximate model counting and almost uniform sampling as implemented in $\mathsf {ApproxMC3}$ and $\mathsf {UniGen2}$ respectively. In this paper, we proposed five technical improvements, as follows: (1) detaching the clausal representation of XOR constraints from unit propagation, (2) lazy reason generation for XOR constraints, (3) bit-level parallelism for XOR constraint propagation, (4) partial solution extraction only covering the sampling set and (5) solution reuse. These improvements were incorporated into the new framework $\mathsf {BIRD2}$, which led to the construction of improved approximate model counter $\mathsf {ApproxMC4}$ and almost uniform sampler $\mathsf {UniGen3}$. Experiments over a large set of benchmarks from various domains clearly show an improvement in running time and 77 more problems could be solved for counting and 51 more for sampling.

## Footnotes

1 While approximate counting techniques [10] only require 2-wise independent hash functions, hashing-based sampling techniques [6, 9] require 3-wise independent hash functions.

2 PAR-2 score, that is, penalized average runtime, assigns a runtime of two times the time limit (instead of a “not solved” status) for each benchmark not solved by a tool.

3 The phrase *strongly 2-universal* is also used to refer to 2-wise independent as noted by Vadhan in [23], although the concept of 2-universal hashing proposed by Carter and Wegman [4] only required that $\mathsf {Pr}[h(x) = h(y)] \le \frac{1}{2^m}$.

4 DSharp is used because of its ability to handle sampling sets.
